# Circulating Hypoxia Responsive microRNAs (HRMs) and Wound Healing Potentials of Green Tea in Diabetic and Nondiabetic Rat Models

**DOI:** 10.1155/2019/9019253

**Published:** 2019-01-01

**Authors:** Hadeel A. Al-Rawaf, Sami A. Gabr, Ahmad H. Alghadir

**Affiliations:** ^1^Rehabilitation Research Chair, College of Applied Medical Sciences, King Saud University, Riyadh, Saudi Arabia; ^2^Department of Clinical Laboratory Sciences, College of Applied Medical Sciences, King Saud University, Riyadh, Saudi Arabia

## Abstract

Green tea (*Camellia sinensis*) has many biological activities and may promote diabetic wound healing by regulation of circulating hypoxia responsive microRNAs (HRMs) which triggers the wound repairing process in diabetic and nondiabetic wounds. Thus, in this study, the potential effects of green tea extract (GTE) on the expression of miRNAs; miR-424, miR-199a, miR-210, miR-21, and fibrogenitic markers; hydroxyproline (HPX), fibronectin (FN), and nitric oxide (NO) were evaluated in wounds of diabetic and nondiabetic rats. The animals were topically treated with vaseline, 0.6% GTE, and 5%w/w povidone iodine (standard control). HPX, FN, and NO levels and microRNAs, miR-424, miR-210, miR-199a, and miR-21, were estimated in wound tissues using colorimetric, immunoassay, and molecular PCR analysis. In vitro analysis was performed to estimate active constituents and their antioxidant activities in methanolic green teat extract (GTE). Wounds treated with green tea, a dose of 0.6, healed significantly earlier than those treated with standard vehicle and vaseline treated diabetic wounds. Higher expressions of HRMs, miR-199a, and miR-21, and lower expression of HRMs, miR-424 and miR-210, were significantly reported in tissues following treatment with green tea extract compared to standard control vehicle. The tissues also contained more collagen expressed as measures of HPX, FN, and NO and more angiogenesis, compared to wounds treated with standard control vehicle. Diabetic and nondiabetic wounds treated with green tea (0.6%) for three weeks had lesser scar width and greater re-epithelialization in shorter periods when compared to standard control vehicle. Expression of HRMs, miR-199a, miR-21, and HRMs and miR-424 and miR-210 correlated positively with HPX, fibronectin, NO, better scar formation, and tensile strength and negatively with diabetes. In addition to antidiabetic and antioxidant activities of green tea components, GTE showed angiogenesis promoting activity in diabetic wound healing. In conclusion, Camellia sinensis extracts in a dose of 0.6% significantly promote more collagen and fibronectin deposition with higher expression of NO, promoting angiogenesis process via molecular controlling of circulating hypoxia responsive microRNAs: miR-424, miR-210, miR-199a, and miR-21 in diabetic and nondiabetic wounds. Our results support a functional role of circulating hypoxia responsive microRNAs: miR-424, miR-210, miR-199a, and miR-21 as potential therapeutic targets in angiogenesis and vascular remodeling in diabetic wound healing.

## 1. Introduction

A wound is a disruption or an injury of the cellular, anatomical, and functional continuity of a living skin tissue. Based on cause and physiology, wounds are classified into incision, laceration, puncture, acute, and chronic [1–3]. Many biological and molecular processes were involved during wound healing, particularly, cell migration, proliferation, object matrix deposition, and remodeling. In addition, inflammation, wound contraction, re-epithelialization, tissue remodeling, and formation of granulated tissues were significantly reported in healing process [4–6].

In patients with diabetic wounds, impaired rates or prolonged healing periods were reported with the developments of neuropathy complications and severe foot ulcers [6–8]. Thus new trends or molecular based pathways should be addressed to overcome the severity of diabetic wound healing.

Circulatory micro-RNAs, a noncoding short RNAs, reported to control the biological processes of wounds via regulation of the expression of certain gene [9]. These small noncoding RNA molecules were shown to play pivotal roles in wound healing [9]. In wounds, as a result of the change in the state of tissue oxygenation, microRNAs referred to as oxymiRs are significantly expressed as indicators of biological and physiological outcomes of wound healing process [10]. These OxymiRs can be directly affected by changes in tissue oxygenation or indirectly by oxygen-sensitive transcription factors, metabolites. Also, expressions of miRNAs during wounding process are sensitive to other oxygen-sensing pathways such as hypoxia and this case is classified as hypoxymiRs.

In our body, HIF is one of the most important hypoxia sensors and a number of hypoxymiRs are induced by HIF stabilization [11]. The data showed that miR-424 was expressed to stabilize HIF1*α*, while miRs that target the HIF1*α* transcript such as miR-20b, miR-199a, and miR-17~92 are repressed to increase HIF1*α* expression and transcription [12, 13].

HypoxymiRs expression was shown to be significantly increased in chronic nonhealing wounds with greater ischemia/hypoxia. Therefore, hypoxymiRs such as miR-210, miR-21, and miR-203 are the most studied hypoxymiRs with respect to wound healing [14], to identify dysregulated targets for possible therapeutic intervention. Thus, microRNA-based gene silencing plays a critical role in the tissue repair response following wounding, and systemic or local delivery of miRNA inducers or inhibitors may provide a therapeutic potential of miRNAs for nonhealing wounds [15, 16].

Several plant materials such as herbs have been used to treat skin disorders and wounds since ancient times [17, 18]. In ethnobotany, some plant extracts possess curative properties that make them useful for wound healing and treating skin diseases, especially in developing countries with poor medical treatment [19–24]. Thus, in diabetic wounds, biologically active agents of plant origin were the most right options for treating strategies via accelerating healing process and promoting the regeneration of cellular damaged tissues [25–27].

Tea (*Camellia sinensis*) is an important source of antioxidants. Green tea, oolong, and tea leaf are prepared from* Camellia sinensis* using different processing methods. They are totally different in chemical composition [28]. These plants contain numerous phytochemicals such as alkaloids, essential oils, flavonoids, tannins, terpenoids, saponins, and phenoplast compounds which are responsible for the curative and preventive biological activities against many human diseases [29, 30]. In addition to that, tea contains many antioxidant phenolic compounds, particularly flavan-3-ols and Epigallocatechin-3-gallate (EGCG), the most abundant catechins present in large amounts which are responsible for the powerfull antioxidant and inhibitory activities of tea [31]. Besides, small amounts of theaflavins, thearubigins, oxyaromatic acids, and flavonols (myricetin, quercetin, flavones, and acid derivatives (tannins)) were elucidated in tea extracts with antioxidant and inhibitory activities [31, 32]. Green tea as a rich source of biologically active phenolic compounds was shown to have antidiabetic [33, 34], antioxidant [19, 35], antimicrobial [36], and wound healing activities [37, 38].

Therefore, in this study, we hypothesized that green tea and its bioactive constituents may have a potential activity to induce changes in the normal expression levels of a set of miRNAs which triggers the wound repairing process in diabetic and nondiabetic wounds. Thus, the potential effects of green tea extract (GTE) on the expression of miRNAs, miR-424, miR-199a, miR-210, and miR-21, and fibrogenitic markers; hydroxyproline (HPX), fibronectin (FN), and nitric oxide (NO) were evaluated in wounds of diabetic and nondiabetic rats.

## 2. Materials and Methods

### 2.1. Maceration Method for Preparation of Green Tea Extract

For this purpose, 100g green tea leaves were added into an Erlenmeyer containing one litre of ethanol 70% and left for forty eight hours at laboratory temperature. Later, the extract was filtrated through a filter paper and the pulp was squeezed to discharge. Then, the extract was concentrated by a rotary evaporator [39], dried, and mixed with pure vaseline to make a vaseline-based 0.6% ointment [40]. To avoid effect of light on green tea polyphenols all extraction procedures were performed under dim light.

### 2.2. Phytoconstituents Screening of Green Tea Extract (GTE)

Various phytochemical screening tests were performed for the identification of the phytoconstituents present in green tea leaves extracted in ethanol by using previously reported standard methods [41].

### 2.3. Estimation of Total Phenolic (TPC) and Flavonoid Contents (TFC) of Aqueous Green Tea Extract

Total flavonoid (TFC) and phenolic contents (TPC) were estimated spectrophotometrically in green tea extract by using 2% aluminum chloride and diluted Folin-Ciocalteu as reagents [42–44]. The absorbance of the reaction mixtures produced was measured at 430 nm and 725 nm respectively as previously reported [44]. Standard calibrated curves of rutin and Gallic Acid were used to estimate flavonoids and poly phenolic compounds in green tea samples. The data obtained were expressed in mg rutin equivalent (RE) per g for flavonoids and Gallic Acid Equivalents/100 mg for poly phenolic compounds, respectively [42–44]. Epigallocatechin 3-gallate (EGCG) as active constituent was estimated in green tea extract by using HPLC method. EGCG content was estimated in 32% (w/w) of the GTE product and was kept in the dark at −20°C until studied [44–46].

#### 2.3.1. Antioxidant Analysis of Aqueous Green Tea Extracts (AGTE)

DPPH and *β*-Carotene-linoleic acid models were used to estimate free radical scavenging and antioxidant activities of the green tea extract. The data were measured by using spectrophotometric analysis as previously reported [44, 47–49]. Various concentrations from the extracted green tea were prepared in hot water and both radical scavenging and antioxidant activities of green tea against DPPH and *β*-Carotene-linoleic acid were estimated as absorbance at *λ* = 517 nm according to

(1) $$\begin{array}{c} Radical\;\;scavenging\;\;activity\left(\;\%\;\right)=\left[\;\frac{\left(Ac-As\right)}{Ac}\;\right]\times 100 \end{array}$$

(See [44]) where (Ac) is the absorbance of the control and (As) is the absorbance of the sample.

(2) $$\begin{array}{c} Antioxidant\;\;activity\left(\;\%\;\right)=\frac{\left(Abs0-Abst\right)}{\left(Abs*0-Abs{}^{\circ}t\right)}\times 100 \end{array}$$

(See [44]) where Abs0 and Abs*∗* 0 are the absorbance values measured at 0 time of incubation for sample extract and control, respectively. Abst and Abs*∗* t are the absorbance values for sample extract and control, respectively, at t = 120 min [44].

### 2.4. Animals and Study Design

A total of 40 healthy Wistar male rats, weighing (180 -250g), were involved in this study. The animals were housed and subjected to normal feeding, drinking, and health care mechanism according to the guidelines of the Experimental Animal Care Centre, College Of Applied Medical Sciences, King Saud University, Riyadh, Saudi Arabia. The experiment and the procedures were approved by the Ethics Committee of the Experimental Animal Care Society, Rehabilitation Research Chair (RRC), College of Applied Medical Sciences, King Saud Univ., Riyadh, Saudi Arabia. Animals had no history of surgery or infection, and other medical interventions were included in this study. The animals were randomly assigned to five groups (8 rats/group).

### 2.5. Induction of Diabetes

Induction of diabetes was performed in rats following an overnight fasting [50]. Each rat was injected intraperitoneally with normal saline containing (160mg/kg body weight) of freshly prepared alloxan monohydrate, the “classical” diabctogenic agent which accumulates in beta cells as glucose analogues via the GLUT2 glucose transports and induce diabetes via free radical oxidative mechanisms of beta cells. The clinical courses of the diabetes induced by alloxan were similar to other diabetogenic agents such as streptozotocin but not identical. The explanation for these differences may be due to extrapancreatic effects, such as a difference in the renal toxicity among the beta cytotoxins used in promoting diabetes. However, alloxan was reported with its well established nephrotoxic effects.

After 48 hours postalloxan injection, to monitor hyperglycaemia, a drop of whole blood obtained from the tail vein was applied onto glucose autoanalyser (ACCU-CHECK Active® Germany) as mentioned previously [51]. Rats with blood glucose level above 135mg/dl were considered diabetic [52].

### 2.6. Excision Wound Model

Excision wound was created as described previously [53]; five groups of animals each containing eight rats were shaved on the dorsum portion using depilatory cream (Reckitt Benckiser, Inc., UK) and anesthetized using ketamine hydrochloride (50 mg/kg, i.p., body weight) in combination with xylazine hydrochloride (10 mg/kg) of body weight [52]. On shaved dorsal region an impression was made and area of the wound to be created was marked. Using toothed forceps and pointed scissors, a full thickness excision wound with a circular area of 314 mm^2^ and 2 ml in depth was created along the marking, and rats were left undressed to the open environment.

### 2.7. Incision Wound Model

Firstly, all rats were anesthetized using ketamine hydrochloride (50 mg/kg, i.p., body weight) in combination with xylazine hydrochloride (10 mg/kg) of body weight and then paravertebral incision of 6 cm length was made on either side of the vertebral column of the rats using a sharp scalpel through the entire thickness of the shaved skin as previously mentioned [54]. The wound was stitched by means of interrupted sutures placed approximately 1cm apart using black silk surgical thread (number 000) and a curved needle (number11). After stitching, the wound was left undressed and animals were treated daily for 10 days. On the 10^th^ day, all rats were anesthetized, and sutures were removed whereas tensile strength of cured wounds was measured using tensiometer.

### 2.8. Wound Treatments

Animals were divided into five groups (8rats/group) and treated as in the following way: Normal control (GI): rats treated with vaseline,(GII): diabetic rats treated with vaseline, (GIII): normal control rats treated with vaseline plus green tea, (GIV): diabetic rats treated with vaseline plus green tea, and (GV): positive standard control rats treated with betadine 5% w/w povidone iodine cream. Standard drug and vaseline-based 0.6% ointment of green tea extract were applied topically twice daily from the day of the operation until the complete healing. Wound contraction and epithelialization period were evaluated every 4^th^ days after wound formation. At the end of the study, rats were anesthetized and tissue samples were collected from the healed wounds, leaving a 5mm margin of normal skin around the edges of the healed wounds. The samples were stored until reused for both wound healing and biochemical analysis.

### 2.9. Measurement of Wound Contraction and Epithelialization Period

In the excision wound model, wound area was measured by tracing the wound with the help of transparent sheet using millimeter based graph paper on days 0, 4, 8, 12, and 16 for all groups. Wound contraction was measured every 4^th^ days until complete wound healing and represented as percentage of healing wound area [55].

Percentage of wound contraction was calculated taking the initial size of the wound as 100% using the following formula [56]: {% wound contraction = (initial wound area−specific day wound area)/initial wound area×100}. In addition, epithelialization period was calculated as the number of days required for falling off the dead tissue remnants of the wound without any residual raw wound [57].

### 2.10. Tensiometer

The tensiometer as previously reported [58] consists of a 6 x 12 inch wooden board with one arm of 4 inch length, fixed on each side of the possible longest distance of the board. The board was placed at the edge of a table. A pulley with a bearing was mounted on the top of one arm. An alligator clamp with 1 cm width was tied on the tip of the other arm by a fishing line in such a way that the clamp could reach the middle of the board. Another alligator clamp was tied on a longer fishing line with a weighing pan on the other end.

### 2.11. Measurement of Tensile Strength

Tensiometer was used to measure tensile strength of excised tissue as previously estimated [59]. The tensile strength of healed skin wounds represents how much the healed tissues resist to breaking under tension, and it may identify the degree and quality of healing tissues. In our experiment, on the 10^th^ day, all animals were anesthetized by injecting ketamine hydrochloride (50mg/kg, i.p., body weight), the sutures were removed, and the healed tissues were excised from all animals.

### 2.12. Estimation of Fibrogenesis Markers

Fibrogenesis markers, hydroxyproline (HPX), fibronectin (FN), and nitric oxide (NO) were estimated form tissue samples from excised wounds as follows.

### 2.13. Estimation of Hydroxyproline (HPX) Levels

Excised tissues were dried to constant weight using a hot air oven at 60°C and samples were hydrolyzed by adding 5mL of 6N HCl in small sealed Pyrex test tubes for 3 hours at 130°C as previously reported [60]. The hydrolysates were then neutralized to pH 7.0 by 2.5 N NaOH and were subjected to chloramine-T oxidation for 20 min in room temperature. Following 5 min, chloramine T oxidation was terminated by adding 0.4 M perchloric acid. 1 mL of Ehrlich's reagent was added to tubes, shaken, and placed in a 60°C water bath for 20 minutes until color development. Tubes were then cooled in tap water for 5 minutes. The absorbance values of the solutions were determined at 557nm in ultraviolet (Systronics-2203) spectrophotometer. The HPX values were calculated from the L–hydroxyproline standard curve.

### 2.14. Estimation of Fibronectin (FN) Levels

Excised tissues were rinsed in 5-10 mL of ice-cold PBS (0.01mol/L, pH 7.0-7.2) and homogenized by using a glass homogenizer on ice. To further break cell membranes, the resulting suspension was sonicated with an ultrasonic cell disrupter. The homogenates were then centrifuged for 5 minutes at 5000×g and the residue was used to estimate the concentration of fibronectin using immunoassay ELISA kit (ABIN1874233, Atlanta, GA30338, USA) at a wavelength of 450 nm using spectrophotometer. The concentration of fibronectin was then determined by comparing the O.D. of the samples to the standard curve as previously reported [61].

### 2.15. Estimation of Nitric Oxide (NO) Levels

Nitric oxide (NO) was estimated as nitrite, a stable NO oxidation product in the tissue homogenates from excised wounds of all studied treated and nontreated groups. It was assessed in the tissue homogenates via using the Griess reaction method as described previously [44, 62]. Equal volumes of standard (0–100 mol/L Na nitrite) or sample were added to the Griess reagent which consisted of equal volumes of 10 g/L sulfanilamide (in 0.5%, nitrite free H_3_PO_4_) and 1 g/L of naphthylethylenediamine. Nitrite levels were then assessed spectrophotometrically at a wavelength of 570 nm[44, 62]

### 2.16. Assessment of Circulating RNAs

#### 2.16.1. Extraction and Purification of Circulating RNA

“TRIzol reagent (Clontech Laboratories Inc., Mountain View, CA, USA) was used to extract total RNA from serum according to the manufacturer's protocol. cDNA of miR-424a, miR-199a, miR-210 and miR-21 were then synthesized using the Mir-X miRNA First-Strand Synthesis Kit (Clontech Laboratories Inc.)”[63]; then “both the integrity and quantity of total RNA were assessed by using an Agilent 2100 Bioanalyzer (Agilent Technologies). The procedures were performed according to the manufacturer's instructions as previously reported” [63, 64].

#### 2.16.2. Real-Time qPCR of microRNAs

“A ready-made solutions containing the primers and probes for miR-146a, miR-155, and miR-15a (Applied Biosystems, Foster City, CA) and real-time RT–PCR was estimated using an ABI 7300 system (Applied Bio systems)” [63, 64]. “Quantitative real-time polymerase chain reaction (qRT-PCR) of miR-424a, miR-199a, miR-210 and miR-21 were conducted using the Mir-X miRNA qRT-PCR SYBR Kit (Clontech Laboratories Inc.) with the Applied Biosystems 7300 Real-Time PCR System (Applied Biosystems, Foster City, CA, USA)”[62, 63]. “Anormalized [*sic*] U6 snRNA levels as an internal quantitative control and the 2−ΔΔCt system were used to estimate thethe [*sic*] expression levels of miRNAs used. Also, all reactions were run in duplicate to avoid errors and exactly determine cycle threshold mean values for each sample including amplified miRNAs” [65, 66].

### 2.17. Statistical Analysis

The results are expressed as mean± standard deviation (M±SD). The statistical significance was analyzed using one-way analysis of variance (ANOVA) followed by Tukey-Kramer Multiple Comparisons Test. One-way ANOVA was used to examine the mean differences in wound healing between the groups in excision and incision wound models. Spearman correlations were used to assess correlations between wound healing evaluation, angiogenesis, and diabetic parameters. The data were analyzed employing SPSS statistical software version17.0 for Windows. Differences between groups were considered significant at P < 0.05 levels.

## 3. Results

### 3.1. Phytoconstituents Screening

The results showed that methanolic GTE contains approximately 18.98% w/w of active phytoconstituents. Alkaloids, flavonoids, glycosides, tannins, triterpenoids, polyphenols, and carbohydrates were shown to be the most common phytoconstituents in GTE (Table 1).

### 3.2. Total Phenolic (TPC) and Flavonoid Contents

The total amounts of phenolic and flavonoid compounds in GTE were found to be 382.6 ± 2.7 mg of gallic acid equivalents per gram of GTE and 78.6 ± 0.81 mg of quercetin equivalents per gram of GTE, respectively (Table 1). EGCG was the most common polyphenolic compound in GTE (32% w/w).

### 3.3. In Vitro Antioxidant Capacity of Green Tea Extract

The free radical scavenging and antioxidant activities of GTE were evaluated using 2,2-diphenyl-1-picrylhydrazyl (DPPH) assay and *β*-carotene-linoleic acid test, respectively. GTE showed a significant (89.7-97.1%) DPPH free radical scavenging activity. In addition, its antioxidant activity in the *β*-carotene-linoleic acid test was 86.3% (Table 1).

### 3.4. Effect of GTE on Wound Closure and Epithelialization Period

**Figure 1:**
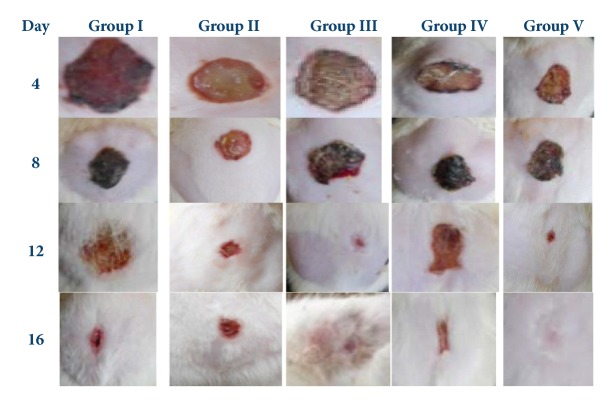
Photographs represent the percentage of wound closer rates on different postexcision days (4th - -16th). Group I: vaseline treated normal control rats, group II: diabetic rats treated with vaseline, group III: normal control rats treated with vaseline plus 0.6 green tea extract, group IV: diabetic rats treated with vaseline plus 0.6 green tea extract, and group V: diabetic rats treated with betadine 5% w/w povidone iodine cream as standard control.

During the course of the treatment, GTE showed its potential effects on wound healing at days 4-16, as shown in Figure 1.

In the diabetic and normal control rats treated with vaseline, the rates of wound contraction were 21.9-41.8% and 25-46.3%, respectively, at days 4-12, and 61.9% and 63%, respectively, at day 16. Also, complete epithelialization and healing were observed at days 21 and 23 in the normal control and diabetic rats, respectively. In addition, a complete scar formation without any raw wound residues occurred at day 20.9 in the normal rats and at day 22.6 in the diabetic rats. After a complete healing, the mean scar area was 99.6 mm^2^ and 94.8 mm^2^ in the normal and diabetic rats, respectively (Table 2).

In green tea treated rats, wound contraction at days 4-12 after treatment with GTE ointment was 34.6-68% in the normal control rats and 35.1-66.8% in the diabetic rats. At day 16, significant wound contraction was observed (normal control rats, 83.1% vs diabetic rats treated with GTE, 81.9%). Complete epithelialization and wound healing were also observed at days 14 and 15 in the normal control and diabetic rats, respectively. In addition, a complete scar formation without any wound residues occurred at day 13.8 in the normal control rats and at day 14.5 in the diabetic rats. The mean scar area after a complete wound healing was 99.8 mm^2^ and 98.6 mm^2^ in the normal control and diabetic rats, respectively.

Green tea treated rats showed faster and efficient wound healing which was comparable to the data obtained from rats treated with betadine 5% w/w povidone iodine cream as control standard (Figure 1 and Table 2). In green tea treated rats, epithelialization process was performed in short time (*P* < 0.01) compared to that present in the control and diabetic rats treated with vaseline and positive standard control.

### 3.5. Effect of GTE on Tensile Strength of the Wounds

**Figure 2:**
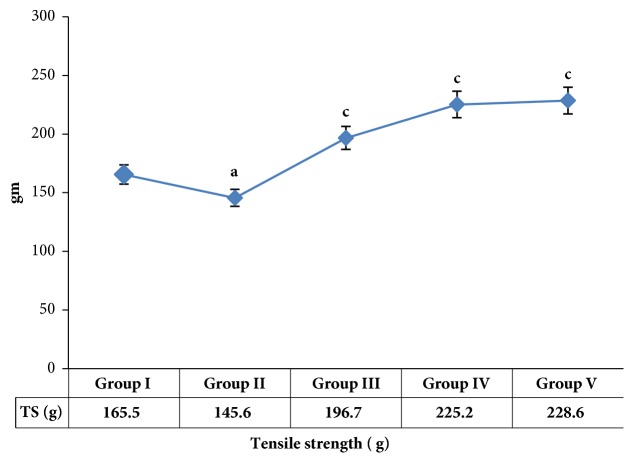
Effect of green tea on tensile strength of skin wounds in incision wound model in Wistar rats. Values are mean ± SEM of 8 rats in each group. ^a^*P* < 0.05, ^b^*P* < 0.01, and ^c^*P* < 0.001 compared to respective control groups (normal and diabetic) and standard. Statistical analysis was done by one-way analysis of variance followed by Tukey-Kramer Multiple Comparisons Test. Group I: vaseline treated normal control rats, group II: diabetic rats treated with vaseline, group III: normal control rats treated with vaseline plus 0.6 green tea extract, group IV: diabetic rats treated with vaseline plus 0.6 green tea extract, and group V: diabetic rats treated with betadine 5% w/w povidone iodine cream as standard control.

The tensile strength of each healed wound was assessed using tensiometer (Figure 2). Compared to the control rats and diabetic rats treated with vaseline, GTE-treated rats showed a significantly higher tensile strength of their wounds (*P* < 0.05 and *P* < 0.001, respectively). This may be related to GTE increasing the viability of collagen fibrils around the wound area to increase tensile strength.

### 3.6. Effect of GTE on Fibrogenesis Markers

The levels of HPX, FN, and NO were significantly (*P*<0.001) higher in GTE- and povidone-iodine-treated rats than in normal control and diabetic rats. Moreover, it was observed that the effects of GTE and povidone iodine were dose-dependent (Table 3). Compared to the normal control rats, the diabetic rats showed a significant decline (*P* < 0.001) in the levels of HPX, FN, and NO as the fibrogenesis markers in their wounds (Table 4).

Correlation analysis showed a significant association (*P* < 0.01) between the levels of HPX, FN, NO, glycated hemoglobin (HbA1c), and wound healing parameters, such as rate of wound closure, scar formation, wound strength, and epithelialization period in normal and diabetic rats treated with GTE (Table 4). The wound healing parameters correlated positively with HPX, FN, and NO levels but negatively with HbA1c levels.

Increases in the levels of HPX, FN, and NO result in an increase in the levels of collagen in healed wounds. This is usually confirmed by an increment in viability or microcirculation of collagen fibrils around the wound area, whereas enhanced wound closure is evidenced in short time by wound strength. The collagen stability or wound strength associated with the various treatments was in the following order: povidone iodine > GTE > Vaseline only in respective order in both diabetic and control rats (Table 2 and Figure 2).

### 3.7. Effect of GTE on miRNAs Expression

**Figure 3:**
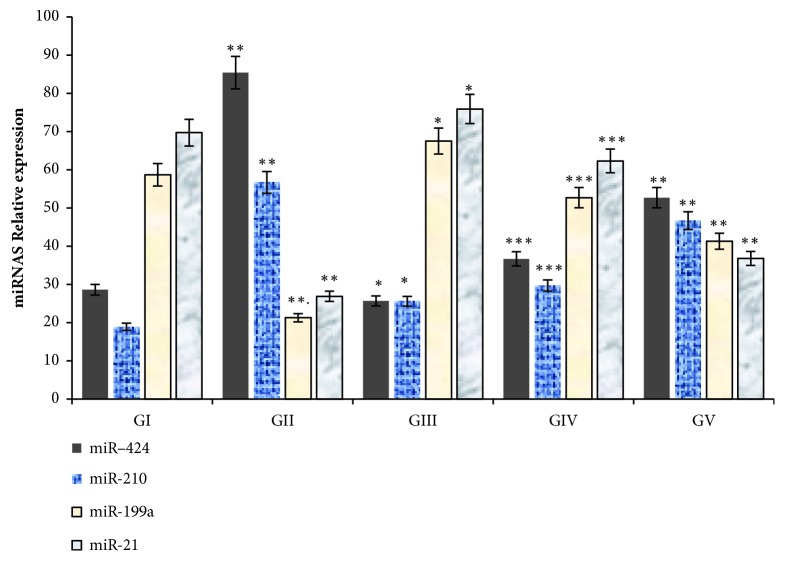
Expression of miRNAs; miR-424, miR-210, miR-199a, and miR-21 in skin tissues of diabetic and nondiabetic wounds treated with green tea extracts and a standard control. Upregulation of miR-424 and miR-210 and downregulation of miR-199a and miR-21 were reported in wounds of diabetic rats compared to control group treated with vaseline only (GI vs GII; r=0.258, P=0.01). Whoever, in diabetic rats treated with vaseline plus 0.6 green tea extract, miR-199a, and miR-21were significantly upregulated (increased), and miR-424 miR-210 were significantly decreased compared to diabetic rats treated with vaseline only (GIV vsGII; r=0.345, P=0.001), respectively. Also, skin wounds of diabetic rats treated with betadine 5% w/w povidone iodine cream as standard control showed an increase in the expression levels of miR-199a and miR-21 and decline in miR-424 and miR-210 compared to respective control groups (GV vs GI; normal or GII; diabetic, r=0.542 p=0.01). In addition, in normal control rats, skin wound treated with vaseline plus 0.6 green tea extract showed considerable upregualtion in the levels of miR-199a and miR-21 and downregulation of miR-424 and miR-210, respectively (GIII vs GI, r=0.287, p=0.05). Statistical analysis was done by one-way analysis of variance followed by Tukey-Kramer Multiple Comparisons Test. Group I: vaseline treated normal control rats, group II: diabetic rats treated with vaseline, group III: normal control rats treated with vaseline plus 0.6 green tea extract, group IV: diabetic rats treated with vaseline plus 0.6 green tea extract, and group V: diabetic rats treated with betadine 5% w/w povidone iodine cream as standard control.

In this study, the expressions of circulating hypoxia responsive microRNAs were estimated in skin tissues of diabetic and nondiabetic wounds treated with either green tea extract or betadine 5% w/w povidone iodine cream as standard control treatment (Figure 3).

Skin wound tissues of diabetic rats showed significant upregualtion (increase) of miR-424, miR-210, and down regulation (decrease) of miR-199a, and miR-21 compared to normal control rats treated with vaseline only (GI vs GII; r=0.258, P=0.01).

In diabetic rats treated with GTE or betadine 5% w/w povidone iodine, significant decrease (p=0.001) in the expression levels of miR-424 and miR-210 and increase (p=0.001) in the levels of miR-199a, and miR-21 were estimated, respectively, as compared to normal and diabetic wounded tissues as shown in Figure 3. When green tea extract at a dose of 0.6 was applied to wounds of nondiabetic control rats, there was a change in the expression levels of miR-424 and miR-210 which significantly decreased compared to miR-199a and and miR-21 levels which significantly increased, respectively, when compared to the values obtained in normal nondiabetic vaseline treated wounds (Figure 3).

The changes in the expression of circulating hypoxia responsive microRNAs, miR-424, miR-210, miR-199a, and miR-21, were shown to be associated with deposition of collagen expressed in terms of hydroxyproline (HPX), fibronectin, and nitric oxide (NO), and wound healing parameters, particularly, wound contraction, closure, epithelialization, tensile strength, and scar formation (Table 5).

Circulating hypoxia responsive microRNAs correlated positively with HPX, fibronectin, NO, wound contraction, closure, epithelialization, scar formation, and tensile strength and negatively with diabetes (Table 5).

## 4. Discussion

Various chemotherapeutic agents have been approved for the management of diabetic wounds. However, most of the drugs do not improve wound healing, as improper maintenance of inflamed tissues, inadequate vessel formation, wound fibrosis [50], and abnormal accumulation of collagen in wounds are still observed, which lead to the formation of unpleasant scars [53]. Moreover, many of these therapeutic agents have been shown to be toxic to keratinocytes, which are needed for wound healing [67–69].

Consequently, many studies have been conducted on herbal drugs to find alternative natural remedies with minimum side effects for wound management [70, 71]. Herbs with potent wound healing activities are reported to have few side effects [72].

Since ancient times, Camellia sinensis has been the most common beverage consumed worldwide for health promotion [73–75]. Besides its antioxidant, anticancer, antiaging, and anti-inflammatory effects, green tea also affects the production and accumulation of collagen during wound healing. It also influences the induction of immune responses [28, 31, 76, 77]. The majority of these biological activities are reported to be due to the polyphenolic compounds, particularly the catechin compounds, in green tea [78, 79].

In the current study, we are trying to evaluate the fibrogentic effect and wound healing activity of methanolic green tea extract in diabetic wounds. The phytochemical analysis performed in this study revealed that considerable amounts of polyphenolic (382.6 mg) and flavonoid (78.6 mg) compounds are contained in 100 g of GTE. In addition, the EGCG content in the extract was found to be up to 32%. However, a different amount of EGCG in GTE has been previously reported [44, 80]. This variation in results might be due to differences in brewing time, type of tea, commercial brands, and the tea-producing country [44, 81]. Secondly, GTE showed considerable antioxidant activity in the DPPH free radical scavenging (86.3%) and *β*-carotene-linoleic acid (89.7 and 97.1% at 500 and 1000 *μ*g/mL, respectively) tests. These results are similar to recently reported data [82, 83]. Our finding that the antioxidant effect of green tea is due to its epigallocatechin content is also consistent with reported data [84, 85].

In this study, delayed wound healing in diabetic rats induced by alloxan monohydrate was significantly improved following treatment with GTE at dose of 0.6% in vaseline-based ointment for three weeks. In diabetic rats treated with GTE, wound contraction, complete scar formation, and epithelialization were significantly improved (P < 0.01) in short time (14.5 days) compared to standard control rats treated with betadine 5% w/w povidone iodine cream (15.8 days) and diabetic nontreated rats (22.6 days), respectively.

The results are in line with those who reported a decline in expression or production of most physiological parameters needed for healing such as growth factors, angiogenic responses, deposition of collagen fibrils, influences on epidermal barrier function, and effects on the quantity of granulated tissues synthesized in wounds [66–69, 86, 87]. Consistent with previous research, poor wound healing observed in our diabetic rats may be related to the fact that higher blood sugar leads to a reduction in the deposition of collagen fibres via minimizing collagen synthesis or enhancing catabolism process of newly synthesized collagen fibrils [88]. Severe complications were significantly associated with poor diabetic wound healing, such as foot ulcers, neuropathy, and ischemia which leads to more than 85% of foot amputations [89–92].

To further estimation of the multiple mechanisms of green tea constituents in wound healing process, hydroxyproline (HPX), fibronectin (FN), and nitric oxide (NO) were estimated as fibrogentic agents in diabetic wounds of excision and incistion wound models. There were significant increases in the levels of HPX, FN, and NO in diabetic wounds treated with GTE.

The increments in the levels of these fibrogenic markers were significantly correlated with accelerated wound closure, scar formation, and lesser time for complete epithelialization. However, lower levels of these biomarkers were reported in diabetic nontreated rats which supports the higher sugar levels suppressing the deposition of collagen fibres via minimizing collagen synthesis or enhancing catabolism process of newly synthesized collagen fibrils [87].

Several cellular and biochemical processes were involved in tissue wound healing. Collagen deposition and remodeling are the final stages of wound healing in the dermis [93]. HPX, FN, and NO levels have been shown to correlate efficiently with wound healing [62–64, 94, 95].

Plasma FN significantly contributes to cell hemostasis, infection control, and enhancing epithelialization and organization of granulated tissues [96, 97]. Previously, it was reported that topical application of plasma fibronectin on diabetic wounds increases fibroblast activity and tumor growth factor-b1 and subsequent improvement in wound healing [69]. Recently, it was reported that application of a small chimeric fibronectin protein to dermal diabetic wounds efficiently enhanced wound closure and increased thickness of granulated wound tissues [70].

Hydroxyproline also was estimated in granulation tissues as biochemical marker for turnover and deposition of collagen and as a most promising indicative marker for the progression of wound healing [87]. Previously, an increment in hydroxyproline content and breaking strength of wounds was achieved following the release of NO needed for the production of collagen in wounded rats treated with NO-donor molsidomine [98].

In the present study, complete wound closure and scar formation were associated with an increase in the levels of HPX, FN, and NO in granulated wound tissues. These may be related to the biological activity of green tea polyphenolic compounds to provide a better synthesis of collagen fibrils in wounds [71] and antimicrobial activity of green tea flavonoids that could prevent the secondary wound infections [75]. Also, the increase in hydroxyproline as marker of collagen deposition in granulated wound tissues following green tea treatment might be due to the action of flavonoids [76].

GTE modulated the production of nitric oxide (NO). ECG in green tea enhances collagen deposition, increases vascular endothelial growth, accelerates angiogenesis, and increases the activities of many enzymes such as iNOS [78, 79, 99, 100]. Furthermore, FN acts as a carrier protein and easily binds to EGCG, which in turn facilitates the healing of diabetic wounds [80]. This may be another reason for the release of FN in granulated tissues besides its function in migration of newly formed collagen and fibroblast activation during diabetic wound healing process [70, 89].

Due to the antifibrotic and anti-inflammatory activities of EGCG, collagen production and FN levels are regulated to prevent the overproduction of extracellular matrix, which ensures that a uniform scar is formed at the end of epithelialization [95, 96, 99]. In addition, previous studies reported that the initial stages of normal wound healing need for a production and deposition of collagen fibres, whereas it plays a pivotal role in preserving both structural integrity and tissue function during fibrogenesis [101, 102]. In addition, HPX, FN, and NO were shown to be associated with normal re-epithelialization and remodeling for wounded tissues [103–109].

Also, green tea contains phenolic compounds especially; EGCG increases the levels of vascular endothelial growth factor, accelerates vessel formation, enhances the production of NO and cyclooxygenase, and subsequently affects wound healing quality by causing complete scar formation [109]. Moreover, it is reported that suitable expansion of the vascular tissues around wounds results in nourishment of tissue cells and improved wound healing [110, 111].

Angiogenesis process was shown the most important step in wound healing process, whereas new blood vessels are formed from pre-existing ones [112], which are significantly required for good wound healing process. In this process, new granulated tissues were synthesized to supply newly healed tissues with oxygen and nutrients [113].

Recently, a variety of miRNAs have been involved in angiogenesis [114, 115]. Studies have shown that angiogenesis was significantly associated with the Hypoxia responsive microRNAs (HRMs) induced by HIF1-*α*under hypoxia in tumor [116–118] and chronic wound healing process [9–11, 119].

Thus, in this study we investigated the potential effects of green tea extract in promoting angiogenesis during wound healing process in diabetic and nondiabetic wounds. Circulating hypoxia responsive microRNAs, miR-424, miR-210, miR-199a, and miR-21, were estimated as molecular markers of angiogenesis in skin wounded tissues following treatment with green tea at a doses of 0.6%. Significant decrease (p=0.001) in the expression levels of miR-424, miR-210, and increases (p=0.001) in the levels of miR-199a, and miR-21 were reported in skin wound tissues of diabetic and nondiabetic rats compared to nontreated diabetic and normal wounds (vaseline ointment only).

In skin wounds treated with green tea at doses of 0.6%, circulating hypoxia responsive microRNAs, correlated positively with deposition of collagen expressed in terms of hydroxyproline (HPX), fibronectin, NO, wound contraction, closure, epithelialization, scar formation, tensile strength, and negatively with diabetes.

Previous research studies reported that wound biology was significantly controlled by the expression of certain genes which are largely under the regulation of naturally occurring circulatory micro-RNAs. These are small noncoding RNA molecules which play pivotal roles in wound healing [9].

In wounds, as a result of the change in the state of tissue oxygenation, microRNAs referred to as oxymiRs are significantly expressed as indicators of biological and physiological outcomes of wound healing process [10]. These OxymiRs can be directly affected by changes in tissue oxygenation or indirectly by oxygen-sensitive transcription factors, metabolites.

Also, expressions of miRNAs during wounding process are sensitive to other oxygen-sensing pathways such as hypoxia and this case are classified as hypoxymiRs. In our body, HIF is one of the most important hypoxia sensors and a number of hypoxymiRs are induced by HIF stabilization [11]. The data showed that miR-424 was expressed to stabilize HIF1*α*, while miRs that target the HIF1*α* transcript such as miR-20b, miR-199a, and miR-17~92 are repressed to increase HIF1*α* expression and transcription [12, 13].

Several studies based up on natural products, containing active compounds particularly, triterpenes, alkaloids, flavonoids, and phenolic biomolecules, promote wound healing by enhancing more phases of healing process, including coagulation, inflammation, fibroplasia, collagenation, epithelialization, and wound contraction [120–122]. Tea has been reported to contain relatively high levels of flavonoids, including catechins and other polyphenols which significantly played a role in wound healing and other complications of human diseases such as cancer [123, 124].

Although green tea active compounds, especially its epicatechin-derived polyphenolic components, showed antiangiogenesis in cancer models [125, 126], wound tissues treated with green tea (*Camellia sinensis*) at doses of 200 and 400 mg/mL showed less inflammatory cells and more both collagen deposition and angiogenesis compared to wounds treated with standard control vehicle [94]. Their data confirmed our results that green tea promotes angiogenesis process via molecular regulation of miR-424, miR-210, miR-199a, and miR-21 in skin wound tissues. Also, like our results, previously reported studies showed that green tea increases the deposition of HPX and FN and promotes increasing NO levels in wound healing [62–64, 93, 94]. Plasma FN significantly contributes to cell hemostasis, infection control, and enhancing epithelialization and organization of granulated tissues [94, 95, 125, 126].

The expression of the components of extracellular matrix (HPX, FN) following treatment with green tea significantly is considered as a scaffold to cell migration, a reservoir, and modulator of the release of growth factors such as FGF2 and TGF-*β* and finally aids in normal growth and maintenance of vessels by angiogenesis process [95, 96].

In conclusion, Camellia sinensis extracts in a dose of 0.6% significantly promote more collagen and fibronectin deposition with higher expression of NO, promoting angiogenesis process via molecular controlling of circulating hypoxia responsive microRNAs: miR-424, miR-210, miR-199a, and miR-21 in diabetic and nondiabetic wounds. Our results support a functional role of circulating hypoxia responsive microRNAs, miR-424, miR-210, miR-199a, and miR-21, as a potential therapeutic targets in angiogenesis and vascular remodeling in diabetic wound healing.

## Figures and Tables

**Table 1:** Phytoconstituents screening and antioxidant activity of green tea extract (GTE mg/100mg).

Item	GTE mg/100mg
Percentage yield	18.98%
Phytochemical screening (+/-):	
Alkaloids	+
Flavonoids	+
Tannins	+
Glycosides	+
Triterpenoids	+
Carbohydrates	+
Steroids	-
Saponins	-
Phytochemical constituents (M ± SD)	
Total polyphenolic content^1^	382.6± 2.7
Total flavonoid content^2^	78.6± 0.81
Epigallocatechin 3-gallate (EGCG)	32% (w/w)
Antioxidant capacity	
Radical scavenging activity (ΒCLA; %)	
At cons. of 500 *μ*g/mL	89.7%
At cons. 1000 *μ*g/mL	97.1%
Total antioxidant activity (DPPH; %)	86.3%

(+/-) presence or absence of phytoconstituents; phytochemical constituents represent as mean ± SD (*n* = 3). ^1^Expressed as mg of gallic acid equivalents (GAE)/g of the dry extract. ^2^Expressed as mg of quercetin equivalents (QE)/g of the dry extract.

**Table 2:** Effect of green tea extract on % wound contraction and epithelialization period of wound in excision wound model.

Group	% wound contraction					Epithelialization period (days)
	4^th^ day	8^th^ day	12^th^ day	16^th^ day	Scar area (mm^2^)	
Group I	186.8 ± 0.75	142.2± 0.98 (25%)	65.9 ± 2.56 (46%)	38.7 ±1.23 (63%)	99.6±3.9	20.9 ± 0.65
Group II	192.2 ± 0.39^a^	151±3.4^a^ (21.9 %)	85.7± 0.98^a^ (41.8 %)	55.6 ±2.7 (61.9 %)^a^	94.8±5.4^a^	22.6 ± 0.45^b^
GroupIII	188.6 ± 0.28	132.3±1.5 (34.6%)^a^	45.9 ± 1.37 (68 %)^b^	28.9 ±1.3 (83.1%)^c^	99.8±2.5^b^	13.8± 0.23^b^
GroupIV	194.6 ± 1.2	128 ± 1.4 (35.1 %)^a^	56.1± 0.98 (66.8%)^b^	22.8 ±0.3 (81.9 %)^c^	98.6±1.8^b^	14.5 ± 0.28^b^
Group V	193.9 ± .98	168 ± 0.45 (16.1 %)	75.3 ± 1.2 (43.5 %)	22.8 ±0.3 (78.6%)	92.3±3.9	15.8 ± 1.4

^a^P < 0.05, ^b^P < 0.01, and ^c^P < 0.001. All values are represented as mean ± SD, *n* = 8 animals in each group. Data were analyzed by one-way ANOVA, followed by Tukey-Kramer Multiple Comparisons Test. Group I: vaseline treated normal control rats, group II: diabetic rats treated with vaseline, group III: normal control rats treated with vaseline plus 0.6 green tea extract, group IV: diabetic rats treated with vaseline plus 0.6 green tea extract, and group V: standard control rats treated with betadine 5% w/w povidone iodine cream.

**Table 3:** Effect of green tea on dry granulation tissue, protein, hydroxyproline, fibronectin, and nitric oxide (NO) in excision wound model.

Group	Dry weight of tissues (mg)	Protein (mg/g dry tissue)	Fibrogenesis markers		
			Hydroxyproline (*μ*g/100mg tissues)	Fibronectin (ng/mL)	NO (nM/mg protein)
Group I	365.3 ± 12.3	56.7 ± 1.9	168.2 ± 13.7	39.4 ± 1.9	61.6 ± 6.1
Group II	291.5 ± 16.5^c^	48.7 ± 3.7^c^	146.3 ± 9.4^c^	28.1 ± 2.9^c^	48.5 ± 5.8^c^
Group III	536.9 ± 28.5^a^	68.3 ± 4.6^a^	235.1± 12.7^c^	62.4 ± 3.8^c^	81.4 ± 2.9^c^
Group IV	615.2 ± 12.3^b^	75.1± 1.8^b^	285±16.8^c^	89.4 ± 5.7^c^	96.8 ± 1.6^c^
Group V	456.3 ± 11.4	70.5 ± 2.8	198.6±6.7	58.2 ± 1.7	79.6 ± 2.5

Values are mean ± SEM of 8 rats in each group. ^a^*P* < 0.05, ^b^*P* < 0.01, and ^c^*P* < 0.001 compared to respective control groups (normal and diabetic) and standard. Statistical analysis was done by one-way analysis of variance followed by Tukey-Kramer Multiple Comparisons Test. Group I: vaseline treated normal control rats, group II: diabetic rats treated with vaseline, group III: normal control rats treated with vaseline plus 0.6 green tea extract, group IV: diabetic rats treated with vaseline plus 0.6 green tea extract, and group V: standard diabetic rats treated with betadine 5% w/w povidone iodine cream.

**Table 4:** Correlation between the rates of wound closer, scar formation, epithelialization period, tensile strength, and fibrogentic markers in diabetic and nondiabetic rats treated with green tea extract.

Item	wound closer (% contraction)	Scar formation (mm2)	Epithelialization period (Days)	Tensile strength (g)
HPX (*μ*g/g of tissues)	0.125^b^	0.367^b^	0.124^b^	0.151^b^
Fibronectin (ng/mL)	0.53^b^	0.48^b^	0.98^b^	0.75^b^
NO (nM/mg protein)	0.257^b^	0368^b^	0.215^b^	0.134^b^
Diabetes (HbA1c)	-0.13^b^	-0.38^b^	-0.46^b^	-0.56^b^

Data presented as coefficient (*R*); ^a^significance at <0.01; ^b^significance at <0.001.

**Table 5:** Correlation between circulating hypoxia responsive microRNAs (HRMs) expression with fibrogentic markers (HPX, NO, and fibronectin), diabetic controls, and wound healing of diabetic skin wound tissues of rats following treatment with green tea extracts and betadine 5% w/w povidone iodine cream as a standard control for three weeks.

Variables	Whole cohort (qRT-PCR) of miRNAs concentrations ^(a)^							
	miR-424		miR-210		miR-199a		miR-21	
	R	P	R	P	R	P	R	P
HPX (*μ*g/g of tissues)	0.41	0.01	0.26	0.01	0.54	0.001	0.68	0.001
NO (nM/mg protein)	0.25	0.01	0.34	0.01	0.38	0.001	0.54	0.001
Fibronectin	0.58	0.01	0.65	0.01	0.43	0.001	0.64	0.001
Diabetes (HbA1c)	-0.18	0.01	-0.24	0.01	-0.31	0.01	-0.28	0.01
Epithelialization period (Days)	0.36	0.01	0.57	0.01	0.48	0.001	0.89	0.001
wound closer (% contraction)	0.49	0.01	0.37	0.01	0.54	0.001	0.79	0.001
Scar formation	0.86	0.01	0.68	0.01	0.37	0.001	0.95	0.001
Tensile strength (g)	0.41	0.01	0.71	0.01	0.46	0.001	0.84	0.001

^a^Data are R (spearman). *Abbreviations.* HbA1C: glycated hemoglobin A1c, miR: microRNA, and HPX: hydroxyproline

## Data Availability

All data generated or analyzed during this study are presented in the manuscript. Please contact the corresponding author for access to data presented in this study.
